# Quality Assessment of Kumu Injection, a Traditional Chinese Medicine Preparation, Using HPLC Combined with Chemometric Methods and Qualitative and Quantitative Analysis of Multiple Alkaloids by Single Marker

**DOI:** 10.3390/molecules23040856

**Published:** 2018-04-09

**Authors:** Ning Wang, Zhi-Yong Li, Xiao-Li Zheng, Qiao Li, Xin Yang, Hui Xu

**Affiliations:** 1 School of Pharmacy, Key Laboratory of Molecular Pharmacology and Drug Evaluation (Yantai University), Ministry of Education, Collaborative Innovation Center of Advanced Drug Delivery System and Biotech Drugs in Universities of Shandong, Yantai University, Yantai 264005, China; w18363852393@163.com (N.W.); z18766576078@163.com (X.-L.Z.); chloeleeq@126.com (Q.L.); 2 State Key Laboratory of Natural Medicine and Traditional Chinese Medicine Injections, Jiangxi Qingfeng Pharmaceutical Co., Ltd., Ganzhou 341000, China; jxlzy2@qfyy.com.cn; 3 School of Chemistry and Chemical Engineering, Yantai University, Yantai 264005, China

**Keywords:** Kumu injection, alkaloids, nigakinone, quality assessment, qualitative and quantitative analysis of multi-components by single marker (QAMS), high performance liquid chromatography (HPLC)

## Abstract

Kumu injection (KMI) is a common-used traditional Chinese medicine (TCM) preparation made from *Picrasma quassioides* (D. Don) Benn. rich in alkaloids. An innovative technique for quality assessment of KMI was developed using high performance liquid chromatography (HPLC) combined with chemometric methods and qualitative and quantitative analysis of multi-components by single marker (QAMS). Nigakinone (PQ-6, 5-hydroxy-4-methoxycanthin-6-one), one of the most abundant alkaloids responsible for the major pharmacological activities of Kumu, was used as a reference substance. Six alkaloids in KMI were quantified, including 6-hydroxy-*β*-carboline-1-carboxylic acid (PQ-1), 4,5-dimethoxycanthin-6-one (PQ-2), *β*-carboline-1-carboxylic acid (PQ-3), *β*-carboline-1-propanoic acid (PQ-4), 3-methylcanthin-5,6-dione (PQ-5), and PQ-6. Based on the outcomes of twenty batches of KMI samples, the contents of six alkaloids were used for further chemometric analysis. By hierarchical cluster analysis (HCA), radar plots, and principal component analysis (PCA), all the KMI samples could be categorized into three groups, which were closely related to production date and indicated the crucial influence of herbal raw material on end products of KMI. QAMS combined with chemometric analysis could accurately measure and clearly distinguish the different quality samples of KMI. Hence, QAMS is a feasible and promising method for the quality control of KMI.

## 1. Introduction

It is well known that high performance liquid chromatography (HPLC) is one of the most common techniques used for qualitative and quantitative analysis of organic compounds, especially in a complex multi-component system. There are two main methods for simultaneous quantitation of multiple components by HPLC, the normal external standard method (ESM) using multiple reference standards, and qualitative and quantitative analysis of multi-component by single marker (QAMS). Although it indeed has played an important role in many fields of research and production regulation, ESM for quality control and evaluation of multi-component system has become challenging in some cases due to the high cost of some reference standards, complexity and chemical diversity, the instability of detection, and many other uncertainties [1,2,3]. In contrast, QAMS method could remarkably reduce the detection time and the experimental cost, and improve the practicability and make the quality control more effective and comprehensive. Furthermore, using the same reference standard, QAMS assay can usually be performed along with HPLC under the same optimal chromatographic conditions for separation of multiple components. Therefore, a combined chemometric HPLC and QAMS method has attracted increasing interest in the field of quality assessment and control of complex multi-component systems [4,5,6].

Traditional Chinese medicine (TCM) injection preparation is a kind of dosage form developed from traditional decoction of herbal medicine. As a combination of TCM theory and modern production techniques, TCM injections have played a pivotal role in various diseases and first aid treatment owing to their ability to bypass first pass metabolism and direct distribution into blood circulation for rapid therapeutic efficacy. Kumu is a commonly-used herbal medicine with the functions of heat-clearing and detoxification from *Picrasma quassioides* (D. Don) Benn., a perennial plant widely distributed throughout southern China [7]. Kumu injection (KMI), a single TCM preparation made from the dry sticks or stems of Kumu extracted by ethanol and repeated acid-base adjustment, has been widely used to treat various inflammatory and infectious diseases, including cold, upper respiratory tract infection, acute tonsillitis, enteritis, and bacillary dysentery [8]. A wealth of research has demonstrated the good safety and clinical efficacy of this TCM injection, especially for the treatment of diarrhea, acute tonsillitis, acute upper respiratory tract infections, and pneumonia in children [9,10,11,12]. More to the point, KMI has a great advantage against bacterial resistance as an antibacterial agent. 

It has been further demonstrated that alkaloids are the main bioactive components responsible for therapeutic efficacy of Kumu and its preparations for their potent activities against infection and abscesses of the respiratory, digestive, and urinary systems [13,14,15,16,17,18]. Up to now, more than sixty *β*-carboline and canthinone alkaloids have been isolated from *P. quassioides*, including nigakinone (5-hydroxy-4-methoxycanthin-6-one), 4,5-dimethoxycanthin-6-one, 3-methylcanthin-5,6-dione, *β*-carboline-1-carboxylic acid, *β*-carboline-1-propanoic acid, and so on. Thus, comprehensive qualitative and quantitative analysis on multiple alkaloids is of great importance for quality assessment and control of Kumu and its products. Several qualitative and quantitative analytical methods have been developed for quality assessment of KMI, such as gravimetry, thin layer chromatography (TLC), and HPLC [8,19,20,21]. However, all these methods suffer from either low resolution, low sensitivity, or identification of rather few marker constituents, and are inadequate for revealing the complex chemical profile. Recently, HPLC multi-component quantitative analyses of alkaloids in KMI have been reported [21,22,23]. The major drawback of these quantitative methods by ESM is the application of multiple reference substances, and innovative methods with capability for quantitative and qualitative analysis of multi-components are urgently needed. The present research was aimed to develop an innovative technique for quality assessment of KMI using HPLC combined with chemometric and QAMS methods, which would provide both qualitative and quantitative insight for such a complex multi-component system with a single reference standard under the same optimal chromatographic conditions.

## 2. Results and Discussion

### 2.1. The Establishment of Analytical Protocol

#### 2.1.1. System Suitability and Chromatographic Peaks Identification

**Figure 1:**
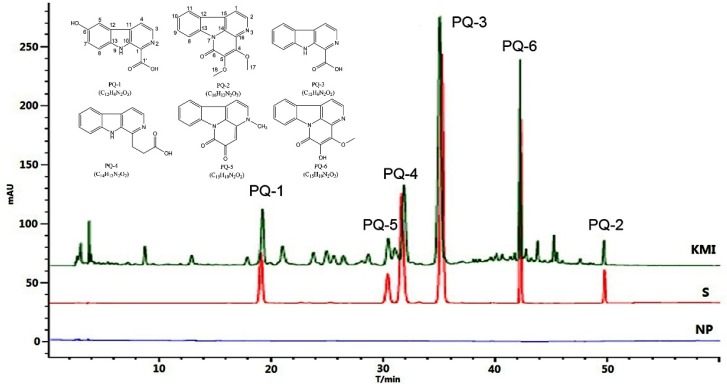
Chromatograms of Kumu injection (KMI) and the mixture of six reference standards. NP: the negative preparation; S: reference standards.

On the basis of the stable baseline, non-peak interference, and maximum absorption, a detection wavelength of 254 nm (Supplementary Figure S1) was selected for the quantitative analysis of the six components, including 6-hydroxy-*β*-carboline-1-carboxylic acid (PQ-1), 4,5-dimethoxycanthin-6-one (PQ-2), *β*-carboline-1-carboxylic acid (PQ-3), *β*-carboline-1-propanoic acid (PQ-4), 3-methylcanthin-5,6-dione (PQ-5), and nigakinone (PQ-6, 5-hydroxy-4-methoxycanthin-6-one). Gradient elution was developed for the effective separation of Kumu injection. Under these chromatographic conditions described in Section 3.4, a good separation was achieved within 55 min for Kumu injection and the standard mixture. The chromatograms are shown in Figure 1. The system suitability for HPLC was evaluated via peak signal to noise ratio, theoretical plate number, resolution, and tailing factor for all the six analytes (Supplementary Table S1). The excellent resolution efficiency of the present HPLC system was demonstrated by fairly high resolution (all up to 1.5) and theoretical plate number (30 711–744 241). Moreover, all the SNRs were more than 1000, and the tailing factors ranged from 0.81 to 1.19, an acceptable range of peak fronting or tailing for HPLC quantitation, indicating that the present HPLC system was suitable for simultaneous determination of all these analytes. The component chosen as the internal referring substance should be stable, easily obtainable, and have a clear pharmacological effect or related to clinical efficacy. Therefore, nigakinone (PQ-6), one of the most abundant and active alkaloid in *P. quassioides* and its preparations [15,16], was used as a quality indicator and an internal referring substance for QAMS of KMI. The RRTs of the other analytes were calculated and used for peak identification in the obtained chromatogram. Under the optimal HPLC conditions in the present study, typical RRT values (mean ± SD) were 0.45 ± 0.02 for PQ-1, 0.72 ± 0.02 for PQ-5, 0.75 ± 0.02 for PQ-4, 0.83 ± 0.01 for PQ-3, and 1.18 ± 0.01 for PQ-2, respectively.

#### 2.1.2. Method Validation

Using S1 as the test sample, the method validation of QAMS was performed on specificity, stability, precision, repeatability, reproducibility, linearity, and accuracy for all the six analytes. PQ-6, the alkaloid with a fairly high abundance and a variety of definite bioactivities responsible for the therapeutic efficacy of *P. quassioides* and its preparations was employed as a reference substance for analysis of KMI. As shown in Figure 1, all the six alkaloids in KMI displayed almost the same chromatographic features as those in standard solution, and the negative control preparation (NP) provided no obvious peak under the same chromatographic conditions, which indicated the satisfactory specificity of the present method.

The stability test was conducted by a five-time analysis of the same KMI sample S1 at different time points (0, 3, 6, 12, and 24 h) after solution preparation at room temperature. The precision and repeatability were evaluated according to the assay of six successive injections and six duplicates of S1, respectively. Meanwhile reproducibility was appraised with the assay of twelve duplicate samples of S1 under the same optimal chromatographic conditions by different instruments and analysts in different laboratories. Relative standard deviations (RSDs) of the relative retention times (RRTs) and relative peak areas (RPAs) of all the analytes were used for evaluation. As to the accuracy investigation, a recovery test was conducted with conventional standard addition method at different concentration levels. The recoveries of all the six analytes averaged 98.3% to 100.1% with the RSDs (*n* = 6) less than 1.4%. As shown in Table 1, all the results demonstrated the present method met the demands of QAMS for stability, precision, repeatability, reproducibility, and accuracy.

The calibration curve was constructed for the six analytes using the series of mixed standard solutions with gradient concentration. Then peak area (*y*) versus concentration (*x*, μg·mL^−1^) data were plotted to give a calibration curve (*y* = *ax* + *b*) by unweighted least-square linear regression analysis, and the linearity, limit of detection (LOD), and limit of quantification (LOQ) were assessed. As shown in Table 2, the correlation coefficient of more than 0.998 indicated satisfactory linearity for all the analytes, and the calibration curve could be utilized for quantitative analysis in the given concentration range.

#### 2.1.3. Assessment of the QAMS Method

In order to assess the QAMS method for simultaneous determination of the major alkaloids in KMI, the relative correction factors (RCFs, *f_x_*) of the co-existing components were firstly determined according to the ratio of the peak areas and the ratio of the concentration between the analyte and PQ-6 as described in Section 3.5. As shown in Table 3, the RCFs averaged 2.162 for PQ-1, 1.335 for PQ-2, 0.737 for PQ-3, 0.654 for PQ-4, and 0.974 for PQ-5, respectively, and the RSDs were 1.8–3.3%, suggesting RCFs of these analytes were fairly constant in the experimental concentration ranges. Although analyte concentration may be one of the major influencing parameters for the determination of RCF, it might vary with the variations in other experimental conditions such as instrument, column, analyst, or environment conditions [3]. Thus, the robustness test was further conducted to investigate the influence of other parameters on RCF determination. With the RSDs ranging from 0.8% to 1.2%, the results shown in Table 4 clearly demonstrated that the present method was durable for the determination of RCFs, and these RCFs could be reliable for QAMS determination of PQ-1 through PQ-5. Then, all the analytes in 20 KMI samples were quantitated by ESM and the QAMS method based on the determination of RCFs, respectively. As shown in Table 5, there were no significant differences between the data from QAMS and ESM method according to paired *t*-test, which indicated that the present QAMS method was reliable for simultaneous quantitation of the alkaloids PQ-1 through PQ-6 in KMI.

### 2.2. Quality Evaluation of KMI Based on Six Components

#### 2.2.1. Concentrations of the Six Components in KMI Samples

The results from QAMS determination of 20 batches of KMI samples showed the mean contents of 15.89 μg·mL^−1^, 6.51 μg·mL^−1^, and 4.01 μg·mL^−1^ for the canthinone alkaloids PQ-6, PQ-5, and PQ-2, and 31.25 μg·mL^−1^, 21.34 μg·mL^−1^, and 13.80 μg·mL^−1^ for the *β*-carboline alkaloids PQ-3, PQ-1, and PQ-4, respectively (Table 5). It is obvious that PQ-6 is one of the most abundant canthinone alkaloids in KMI, thus is well-deserved as a reference substance and index for quality assessment and control of KMI.

Meanwhile, obvious inter-batch content variations could be found for all these alkaloids with the RSDs ranging from 12.1% to 24.9%, and these six alkaloids in total averaged 92.71 μg·mL^−1^ in KMI with a RSD of 13.2% for the 20 batches of samples. The data in Table 5 presented differences among various samples. To show the clear classification of the KMI samples, the QAMS method with chemometrics analysis was performed in the subsequent analyses.

#### 2.2.2. Hierarchical Cluster Analysis (HCA)

**Figure 2:**
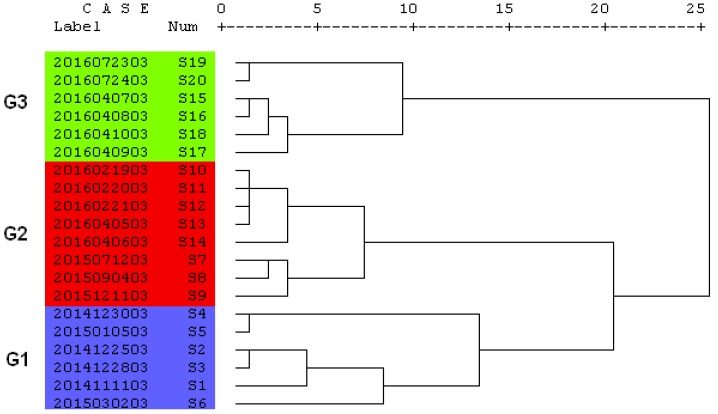
Dendrogram of six chemical compositions for 20 KMI samples. G1: group 1; G2: group 2; G3: group 3.

Using the contents of six alkaloids from 20 KMI samples as the clustering variable, the HCA of the standardized data was performed with the Euclidean distance and Ward’s linkage clustering by SPSS software. The dendrogram shown in Figure 2 illustrated clearly that all the samples could be categorized into three groups with Group 1 (G1) containing S1 through S6, Group 2 (G2) containing S7 through S14, and Group 3 (G3) containing S15 through S20, respectively. It was interesting that such grouping was dependent on the difference in production period, which was reflected by the successive batch numbers of those test samples in the same group. Further investigation revealed that three batches of raw herb materials with different sources had been utilized to produce these twenty batches of KMI (Hailixin^®^) in different time periods, indicating that the quality of TCM preparations varied very much depending on the raw materials, especially under a stable production process. Therefore, it is of great importance for TCM preparations to control the raw herb materials from the source, and the good agriculture process (GAP) and quality assessment system are the most urgent tasks, especially for those wild medicinal herbs such as *P. quassioides*. The results showed that HCA can classify the similarity of KMI on the basis of the contents of the six components. However, HCA failed to clearly indicate which group had a high quality. Therefore, radar plot analysis was used in the following quality analysis. 

#### 2.2.3. Radar Plot Analysis

**Figure 3:**
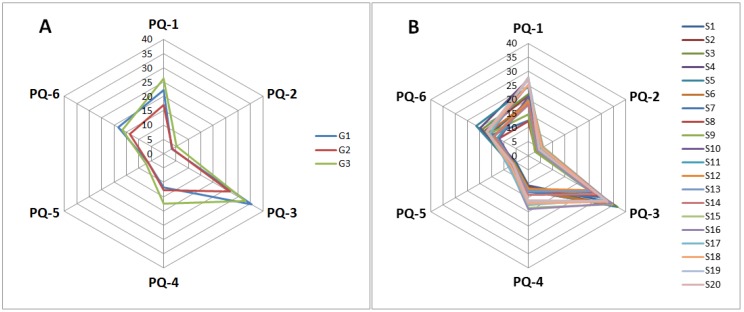
Radar plots showing the difference of geographical origins in terms of six components in various KMI samples; (**A**) G1, G2, and G3. (**B**) The distribution of the chemical composition of 20 KMI samples.

A radar plot was used to assess the quality of KMI samples because of its simple, rapid, and routine discrimination. For ease of comparison, the radar plot was employed to preliminarily classify KMI samples on the basis of the contents of the six components. Radar plot analyses were conducted on 20 KMI samples. Figure 3A shows the means of the six components in G1–G3 in the HCA. As shown, the distributions of the six components of the KMI samples from various groups exhibited different characteristic patterns. The samples from G2 had a distinctly lower content of the six components and were easily discriminated compared with the KMI samples from the other two groups. The differences between three groups of PQ-1, PQ-3, and PQ-6 were large. These three components may be main factors causing the batch-to-batch variation. Therefore, radar plot analysis could distinguish the quality of the different KMI samples. As shown in Figure 3B, the distribution of the chemical composition patterns displayed similar characteristics. This finding indicated that the KMI samples from the various localities had similar features because of species heredity. However, visual measurement was the only result received by radar plot analysis, and the lack of clear indicators describing the exact distinctions reduced the dependability of the results. Therefore, Principal component analysis (PCA) was utilized in the following studies.

#### 2.2.4. Principal Component Analysis (PCA)

**Figure 4:**
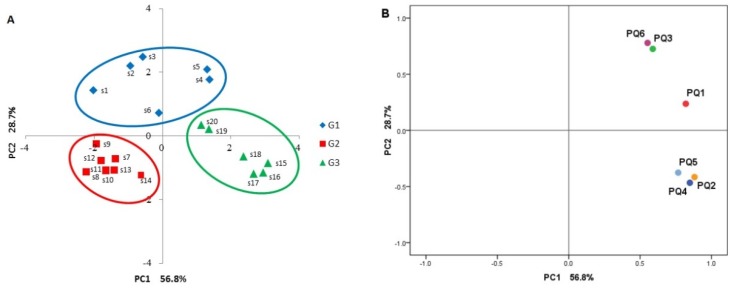
Score plot of 20 KMI samples by PC1 and PC2 from PCA. (**A**) Scatter diagram of KMI samples from three different groups (G1, G2, and G3); (**B**) Loading plot of six components for PC1 and PC2.

The contents of six alkaloids in the KMI samples were further analyzed by PCA, a data mining method for feature extraction and dimensionality reduction to provide an overview of class separation, clustering, and outliers. Two principal components (PCs) that could account for 85.5% of the total variance were extracted with PC1 for 56.8%, and PC2 for 28.7%, respectively. According to the score plots of PC1 and PC2, all the KMI samples were clustered, and the result was consistent with that of HCA (Figure 2 and Figure 4A). Factor analysis was further performed to describe the dominant components that mainly contributed to the variances of all samples, and the loading result is displayed in Figure 4B. It was clearly illustrated that PQ-1 through PQ-6 had a fairly high loading on PC1, while PQ-1, PQ-3, and PQ-6 had a relatively high loading on PC2. Thus, the three alkaloids, namely PQ-1, PQ-3, and PQ-6, may be the main factors causing batch-to-batch variation of KMI samples, and could be considered as major markers responsible for PCA classification. These findings were consistent with those from the radar plots.

Further investigation revealed that such content variation was closely related to the production period of KMI, which was highly consistent with the results from HCA as described above and suggested that the quality of raw herb material *P. quassioides* may have a great influence on the end preparations of KMI. Therefore, an effective quality control and improvement system of Kumu injection should pay great attention to the quality assessment and control of its raw herbs.

## 3. Materials and Methods

### 3.1. Materials and Reagents

Twenty batches of KMI samples (Hailixin^®^, approval number Z36021083) along with negative preparations were supplied by Jiangxi Qingfeng Pharmaceutical Co., Ltd. (Ganzhou, China) (Supplementary Table S2). Six alkaloid components with purity more than 98% were isolated from the alcohol extract of stems of *P. quassioides* by using silica gel chromatography and crystallization in acetone [24] including 6-hydroxy-*β*-carboline-1-carboxylic acid (PQ-1), 4,5-dimethoxycanthin-6-one (PQ-2), *β*-carboline-1-carboxylic acid (PQ-3), *β*-carboline-1-propanoic acid (PQ-4), 3-methylcanthin-5,6-dione (PQ-5), and nigakinone (PQ-6). Their chemical structures were unambiguously elucidated by using comprehensive spectroscopic analysis (Supplementary Figures S1 and S2) and comparing with literature data [25,26] (Figure 1), and the spectral data are as follows.

PQ-1: MS *m*/*z*: 229.0609 [MH]^+^. ^1^H-NMR (in MeOD, 400 MHz) *δ*: 7.90 (1H, d, *J* = 5.20 Hz, H-3), 8.12 (1H, d, *J* = 5.20 Hz, H-4), 7.47 (1H, d, *J* = 8.24 Hz, H-5), 9.19 (1H, s, H-6), 7.09 (1H, d, *J* = 7.96 Hz, H-7), 7.66 (1H, d, *J* = 7.88 Hz, H-8), 11.46 (1H, s, H-9). ^13^C-NMR (in MeOD, 100 MHz) *δ*: 146.00 (C-1), 133.77 (C-3), 116.96 (C-4), 105.47 (C-5), 151.08 (C-6), 119.03 (C-7), 113.58 (C-8), 135.00 (C-10), 120.40 (C-11), 130.33 (C-12), 135.92 (C-13), 168.04 (C-1′); PQ-2: MS *m*/*z*: 281.0921 [MH]^+^. ^1^H-NMR (in MeOD, 400 MHz) *δ*: 7.92 (1H, d, *J* = 4.96 Hz, H-1), 8.83 (1H, d, *J* = 5.00 Hz, H-2), 8.64 (1H, d, *J* = 8.20 Hz, H-8), 7.69 (1H, t, *J* = 7.8 Hz, H-9), 7.50 (1H, t, *J* = 7.68 Hz, H-10), 8.07 (1H, d, *J* = 7.72 Hz, H-11), 4.47 (3H, s, H-17), 4.08 (3H, s, H-18). ^13^C-NMR (in MeOD, 100 MHz) *δ*: 115.64 (C-1), 145.33 (C-2), 152.91 (C-4), 140.01 (C-5), 158.41 (C-6), 117.02 (C-8), 130.82 (C-9), 125.33 (C-10), 122.59 (C-11), 124.76 (C-12), 139.13 (C-13), 128.40 (C-14), 130.06 (C-15), 133.47 (C-16), 61.48 (C-17), 61.40 (C-18); PQ-3: MS *m*/*z*: 213.0658 [MH]^+^. ^1^H-NMR (in MeOD, 400 MHz) *δ*: 8.45 (1H, d, *J* = 5.20 Hz, H-3), 8.48 (1H, d, *J* = 5.20 Hz, H-4), 8.32 (1H, d, *J* = 7.88 Hz, H-5), 7.63 (1H, t, *J* = 7.34 Hz, H-6), 7.32 (1H, t, *J* = 7.52 Hz, H-7), 7.84 (1H, d, *J* = 8.24 Hz, H-8), 11.84 (1H, s, H-9). ^13^C-NMR (in MeOD, 100 MHz) *δ*: 141.92 (C-1), 135.35 (C-3), 118.43 (C-4), 122.01 (C-5), 129.32 (C-6), 120.13 (C-7), 113.06 (C-8), 131.50 (C-10), 119.85 (C-11), 130.72 (C-12), 135.45 (C-13), 165.55 (C-1′); PQ-4: MS *m*/*z*: 241.0969 [MH]^+^. ^1^H-NMR (in MeOD, 400 MHz) *δ*: 8.24 (1H, d, *J* = 5.28 Hz, H-3), 7.95 (1H, d, *J* = 5.28 Hz, H-4), 8.21 (1H, d, *J* = 7.84 Hz, H-5), 7.54 (1H, td, *J* = 7.34 Hz, H-6), 7.32 (1H, t, *J* = 7.44 Hz, H-7), 7.60 (1H, d, *J* = 8.16 Hz, H-8), 11.66 (1H, s, H-9), 2.87 (2H, t, *J* = 7.36 Hz, H-1′), 3.35 (2H, t, *J* = 7.32 Hz, H-2′). ^13^C-NMR (in MeOD, 100 MHz) *δ*: 144.04 (C-1), 137.27 (C-3), 112.84 (C-4), 121.66 (C-5), 127.86 (C-6), 119.22 (C-7), 111.95 (C-8), 134.02 (C-10), 121.01 (C-11), 127.10 (C-12), 140.41 (C-13), 31.32 (C-1′), 28.04 (C-2′), 174.10 (C-3′); PQ-5: MS *m*/*z*: 251.0815 [MH]^+^. ^1^H-NMR (in MeOD, 400 MHz) *δ*: 7.51 (1H, d, *J* = 6.80 Hz, H-1), 8.07 (1H, d, *J* = 6.84 Hz, H-2), 6.02 (1H, s, H-4), 8.46 (1H, d, *J* = 8.20 Hz, H-8), 7.70 (1H, t, *J* = 7.85 Hz, H-9), 7.55 (1H, t, *J* = 7.54 Hz, H-10), 8.24 (1H, d, *J* = 7.76 Hz, H-11), 3.91 (3H, s, H-17). ^13^C-NMR (in MeOD, 100 MHz) *δ*: 104.20 (C-1), 136.62 (C-2), 93.34 (C-4), 170.60 (C-5), 157.40 (C-6), 116.14 (C-8), 130.02 (C-9), 125.61 (C-10), 123.07 (C-11), 120.62 (C-12), 136.95 (C-13), 139.42 (C-14), 124.85 (C-15), 140.50 (C-16), 40.96 (C-17); PQ-6: MS *m*/*z*: 267.0763 [MH]^+^. ^1^H-NMR (in MeOD, 400 MHz) *δ*: 7.51 (1H, d, *J* = 6.80 Hz, H-1), 8.07 (1H, d, *J* = 6.84 Hz, H-2), 6.02 (1H, s, H-4), 8.46 (1H, d, *J* = 8.20 Hz, H-8), 7.70 (1H, t, *J* = 7.85 Hz, H-9), 7.55 (1H, t, *J* = 7.54 Hz, H-10), 8.24 (1H, d, *J* = 7.76 Hz, H-11), 3.91 (3H, s, H-17). ^13^C-NMR (in MeOD, 100 MHz) *δ*: 114.57 (C-1), 145.88 (C-2), 142.68 (C-4), 136.37 (C-5), 157.98 (C-6), 116.68 (C-8), 130.66 (C-9), 125.73 (C-10), 122.67 (C-11), 125.41 (C-12), 138.63 (C-13), 125.96 (C-14), 130.14 (C-15), 134.22 (C-16), 61.01 (C-17).

Methanol and acetonitrile were of HPLC grade and obtained from Fisher Scientific Co. (Fair Lawn, NJ, USA). All other reagents were of analytical grade and purchased from Sinopharm Chemical Reagent Co., Ltd. (Shanghai, China). Ultra-pure water was used in the present study and prepared by a lab purification system (PURELAB Classic UVF, ELGA LabWater, High Wycombe, Buckinghamshire, UK).

### 3.2. Experimental Design

**Figure 5:**
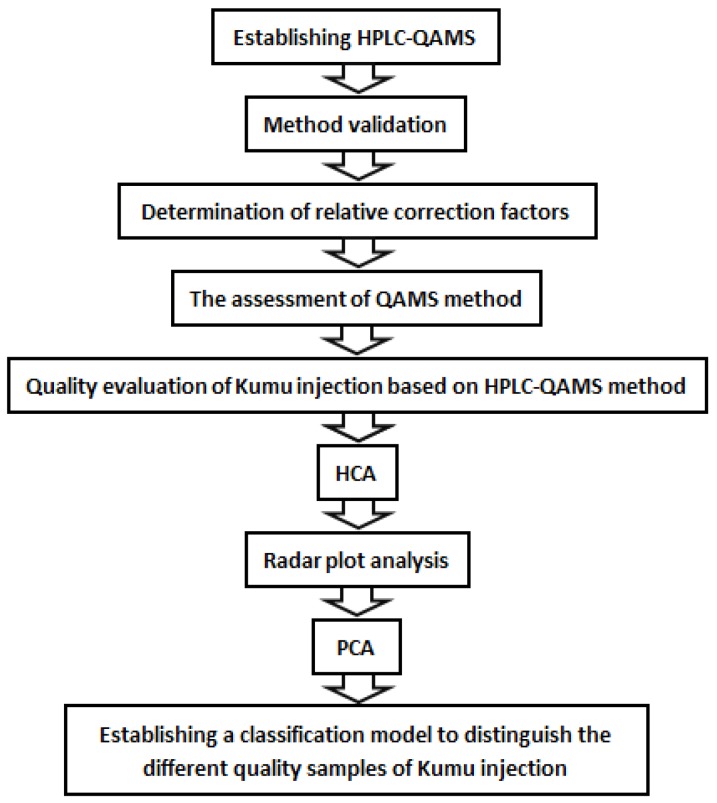
Flow diagram of the experiment.

An accessible HPLC–QAMS method was established for quality evaluation of KMI in the present study. HPLC fingerprints were performed at first to separate the index compositions from KMI. The RCFs then were calculated after systemic method validation for investigation of the stability, precision, repeatability, reproducibility, recovery, and so on. Furthermore, the applicability and feasibility were assessed by paired *t*-test of the results from the QAMS method and those from the ESM. Finally multivariate analyses such as HCA, radar plot analysis, and PCA were conducted for sample classification and quality assessment of KMI based on the contents of six alkaloids (Figure 5).

### 3.3. Preparation of Sample Solutions and Standard Solutions

Sample solution was prepared by filtration of the mixture of three aliquots of KMI through a 0.45 μm membrane before it was injected for HPLC analysis. Stock standard solutions of the six analytes were prepared by dissolving approximately 10 mg of each reference standard respectively into a 10 mL volumetric flask with acetonitrile to volume, mixed, and then diluted with acetonitrile/0.2% aqueous phosphoric acid (7:93, *v*/*v*) to obtain working solutions of each reference standard and the mixed standard for establishing calibration curves. The concentration ranges were 11.0–41.3 μg·mL^−1^ for PQ-1, 1.95–9.75 μg·mL^−1^ for PQ-2, 20.6–62.0 μg·mL^−1^ for PQ-3, 5.22–31.3 μg·mL^−1^ for PQ-4, 2.00–14.1 μg·mL^−1^ for PQ-5, and 2.00–100 μg·mL^−1^ for PQ-6, respectively. All the standard solutions were stored at 4 °C and brought to room temperature before use.

### 3.4. HPLC System and Conditions

HPLC analyses were primarily performed by an Agilent 1100 system composed of a binary gradient pump (G1312A), on-line degasser (G1379A), autosampler (G1313A), column temperature controller, and variable wavelength UV detector (G1314A) coupled with an analytical workstation (Agilent Technologies, Inc., Santa Clara, CA, USA). Analytes were separated on a reverse phase C_18_ column (Phenomenex Gemini C_18_, 250 mm × 4.6 mm, 5 μm). Samples with a volume of 20 μL were injected into the column for separation at a column temperature of 30 °C. At a flow rate of 1.0 mL·min^−1^, the mobile phase composed of solvent mixture of acetonitrile (solvent A) and 0.2% aqueous phosphoric acid (solvent B) was programmed with a linear gradient of solvent A as follows, 0–30 min, 7–13%; 30–50 min, 13–50%; 50–55 min, 50%. An additional 80 min chromatographic run with acetonitrile increasing from 50% to 90% during 55–80 min was performed to obtain a full chromatogram for checking whether all the components could be eluted within 55 min (Supplementary Figure S3). The detection wavelength was set at 254 nm and each sample was injected three times in parallel. A Shimadzu LC-20AT HPLC system (Kyoto, Japan) mainly equipped with a binary gradient pump, and a photo-diode array detector (PDA) was used for chromatographic peak purity test and UV spectrometric determination. An Agilent 1260 HPLC system (Agilent Technologies, Inc., Santa Clara, CA, USA) equipped with a binary gradient pump and a PDA was used for robustness test.

### 3.5. Determination of Relative Correction Factors (RCFs)

The QAMS method is based on the principle of the linear relationship between a detector response and the levels of components within certain concentration ranges [3]. PQ-6 was designated as the reference substance for QAMS. The relative correction factors (RCFs, *f_x_*) of the co-existing components were calculated by the ratio of the peak areas and the ratio of the concentration between the analyte and PQ-6 (Equation (1)), and the quantification could be carried out with both their RCFs and peak areas according to the following equation (Equation (2)).

(1) $$f_{x}\;=\;\frac{A_{s}/C_{s}}{A_{x}/C_{x}}$$

(2) $$C_{x}\;=\;\frac{A_{x}\times C_{s}}{A_{s}}\times f_{x}$$

where *x* and *s* represent the analyte and the internal referring substance PQ-6, respectively, and *f_x_* is the average RCF value of each co-existing component to PQ-6, *A_x_* and *C_x_* denote the peak area and the concentration of the co-existing component in the single standard solution or in the samples, respectively.

### 3.6. Data Analysis

HCA and PCA were performed using the software of SPSS (International Business Machines Corporation, New York, NY, USA) 16.0 to further investigate the difference among the KMI samples. Radar plot analysis was manipulated by using Microsoft Excel 2010.

## 4. Conclusions

In the present study, an innovative technique for quality assessment of Kumu injection, a traditional Chinese medicine preparation, was developed using HPLC combined with chemometric and QAMS methods for the first time. The major bioactive alkaloid nigakinone was applied as reference substance for simultaneous quantitation of six major alkaloids in KMI. The findings provided evidence of the feasibility of the QAMS method using RCFs for simultaneous quantitation of multiple components. Moreover, chemometric combined with QAMS methods could be a powerful and reliable way to provide both qualitative insight and quantitative data for comprehensive quality assessment of complex multi-component systems, especially TCM preparations.

## Figures and Tables

**Table 1:** Results of stability, precision, repeatability, reproducibility, and recovery tests for the QAMS method ^1^.

Analytes	Stability		Precision		Repeatability		Reproducibility		Recovery
	(*n =* 5)		(*n =* 6)		(*n =* 6)		(*n =* 12)		(*n =* 6)
	RSD/%		RSD/%		RSD/%		RSD/%		RSD/%
	RRT	RPA	RRT	RPA	RRT	RPA	RRT	RPA	RPA
PQ-1	0.7	3.1	1.7	1.4	2.2	5.5	3.2	6.0	0.6
PQ-2	0.1	2.1	0.1	2.7	0.1	3.7	0.3	4.6	1.1
PQ-3	0.2	0.6	0.8	3.4	1.0	5.4	2.7	5.2	1.4
PQ-4	1.0	2.0	1.1	3.3	1.7	3.8	3.4	4.8	1.0
PQ-5	0.6	4.4	0.9	3.8	1.4	2.6	2.8	5.4	0.7
PQ-6	0.0	0.0	0.0	0.0	0.0	0.0	0.0	0.0	0.7

^1^ RPA—relative peak area; RRT—relative retention time; RSD—relative standard deviation.

**Table 2:** Typical calibration curves of the six analytes (*n* = 6) ^1^.

Analytes	Regression Equation	R	LOD (μg·mL^−1^)	LOQ (μg·mL^−1^)	Linear Range(μg·mL^−1^)
PQ-1	*y* = 42.681*x* − 59.049	0.9990	0.110	0.404	10.9–40.9
PQ-2	*y* = 65.376*x* − 5.5050	0.9997	0.066	0.241	2.0–9.8
PQ-3	*y* = 119.41*x* − 110.70	0.9998	0.039	0.139	20.6–62.0
PQ-4	*y* = 133.80*x* − 38.727	0.9997	0.033	0.122	5.2–31.3
PQ-5	*y* = 96.928*x* − 45.405	0.9991	0.058	0.188	1.5–14.1
PQ-6	*y* = 86.712*x* − 2.3510	0.9999	0.051	0.186	2.0–100.0

^1^ LOD—limits of detection; LOQ—limits of quantitation. In the regression equation *y = ax + b*, *y* refers to the peak area and *x* refers to the concentration of the analytes (μg·mL^−1^). R is the correlation coefficient of the equation.

**Table 3:** Results of RCFs (*f_x_*) for the five analytes PQ-1 through PQ-5 ^1^.

Concentration Level	PQ-1	PQ-2	PQ-3	PQ-4	PQ-5
C1	2.174	1.296	0.735	0.651	1.009
C2	2.141	1.317	0.720	0.635	1.024
C3	2.204	1.326	0.731	0.653	0.975
C4	2.221	1.382	0.752	0.671	0.975
C5	2.146	1.368	0.753	0.664	0.955
C6	2.084	1.322	0.732	0.648	0.939
Mean	2.162	1.335	0.737	0.654	0.974
RSD/%	2.3	2.5	1.8	1.9	3.3

^1^ RCFs—relative correction factors; *f_x_* is the RCFs value of the other five co-existing components to PQ-6; the values of *C_x_*(*x* = 1–6) in Table denote the concentration of each analyte in mixed standard solutions which used for calibration curves, respectively; RSD—relative standard deviation.

**Table 4:** Results of robustness test for RCFs determination ^1^.

Instrument	Column No.	*f_x_*/PQ-1	*f_x_*/PQ-2	*f_x_*/PQ-3	*f_x_*/PQ-4	*f_x_*/PQ-5
Agilent 1100	1#	2.162	1.335	0.737	0.654	0.974
	2#	2.182	1.353	0.745	0.663	0.990
	3#	2.143	1.325	0.729	0.647	0.969
Shimadzu LC-20AT	1#	2.165	1.337	0.740	0.655	0.972
	2#	2.186	1.352	0.751	0.659	0.987
	3#	2.146	1.325	0.730	0.649	0.967
Agilent 1260	1#	2.160	1.340	0.740	0.655	0.980
	2#	2.185	1.357	0.746	0.660	1.001
	3#	2.144	1.329	0.730	0.651	0.976
mean		2.164	1.339	0.739	0.655	0.980
SD		0.017	0.012	0.008	0.005	0.011
RSD/%		0.8	0.9	1.1	0.8	1.2

^1^ RCFs—relative correction factors; *fx* is the RCFs value of the other five co-existing components to PQ-6; SD—standard deviation; RSD—relative standard deviation.

**Table 5:** Contents of the six analytes in KMI samples determined by ESM and QAMS methods (μg·mL^−1^) ^1^.

Sample No.	PQ-6	PQ-1			PQ-2			PQ-3			PQ-4			PQ-5			Total
	ESM	ESM	QAMS	SD	ESM	QAMS	SD	ESM	QAMS	SD	ESM	QAMS	SD	ESM	QAMS	SD	
S1	15.54	19.45	19.46	0.01	2.77	2.77	0.00	32.81	32.82	0.01	10.51	10.50	0.01	5.07	5.08	0.01	86.18
S2	17.67	21.30	21.31	0.01	2.95	2.95	0.00	36.25	36.27	0.01	11.19	11.18	0.01	5.42	5.44	0.01	94.82
S3	18.93	21.74	21.74	0.01	2.97	2.97	0.00	36.70	36.72	0.01	11.51	11.50	0.01	5.55	5.57	0.01	97.44
S4	19.61	27.19	27.20	0.01	3.91	3.91	0.00	35.95	35.97	0.01	12.51	12.50	0.01	7.05	7.07	0.01	106.27
S5	21.31	24.51	24.52	0.01	4.00	4.00	0.00	35.87	35.89	0.01	12.41	12.40	0.01	6.97	6.98	0.01	105.11
S6	16.38	18.92	18.90	0.02	3.73	3.73	0.00	35.02	35.00	0.01	12.41	12.44	0.02	6.80	6.78	0.01	93.22
S7	14.62	12.49	12.47	0.01	3.27	3.27	0.00	29.20	29.18	0.01	13.12	13.15	0.02	6.93	6.92	0.01	79.60
S8	12.15	12.19	12.21	0.01	2.98	2.98	0.00	28.21	28.19	0.01	12.36	12.36	0.00	6.59	6.59	0.00	74.48
S9	15.71	14.75	14.76	0.00	2.79	2.79	0.00	26.47	26.48	0.01	12.19	12.18	0.01	6.37	6.38	0.01	78.30
S10	11.93	21.11	21.08	0.02	3.61	3.61	0.00	26.22	26.20	0.01	12.39	12.41	0.02	5.90	5.89	0.01	81.12
S11	12.67	19.77	19.74	0.02	3.75	3.75	0.00	25.58	25.56	0.01	12.38	12.40	0.02	5.94	5.92	0.01	80.04
S12	14.00	19.43	19.41	0.02	3.67	3.66	0.00	24.60	24.58	0.01	11.85	11.87	0.02	5.83	5.81	0.01	79.34
S13	13.95	17.89	17.86	0.02	3.89	3.89	0.00	25.31	25.30	0.01	12.83	12.86	0.02	6.18	6.17	0.01	80.02
S14	13.76	18.16	18.19	0.02	4.34	4.34	0.00	27.65	27.64	0.01	14.07	14.07	0.00	6.48	6.48	0.00	84.47
S15	16.83	25.06	25.09	0.02	6.04	6.04	0.00	34.53	34.51	0.01	18.49	18.50	0.00	7.58	7.58	0.00	108.56
S16	15.83	25.23	25.26	0.02	5.73	5.73	0.00	34.24	34.22	0.01	18.96	18.96	0.00	7.71	7.71	0.00	107.73
S17	15.90	27.85	27.86	0.01	5.26	5.27	0.00	31.36	31.38	0.01	17.56	17.55	0.01	8.00	8.02	0.01	105.98
S18	16.98	25.17	25.18	0.01	5.64	5.65	0.00	32.62	32.63	0.01	17.08	17.07	0.01	7.29	7.31	0.01	104.81
S19	17.19	26.89	26.90	0.01	4.47	4.48	0.00	32.22	32.24	0.01	16.33	16.32	0.01	6.39	6.40	0.01	103.53
S20	16.82	27.63	27.65	0.01	4.40	4.41	0.00	32.24	32.26	0.01	15.85	15.84	0.01	6.12	6.14	0.01	103.10
Mean	15.89		21.34			4.01			31.15			13.80			6.51		92.71
RSD/%	15.4		22.7			24.9			13.3			18.7			12.1		13.2
*Sig.* (2-tailed)		0.53			0.36			0.78			0.61			0.19			

^1^ ESM—external standard method, and its content was determined by calibration equation method; QAMS—quantitative analysis multi-components by single marker, and its content was determined by RCFs; SD—standard deviation; *Sig.* (2-tailed)-two paired *t*-test results; Total-the sum of the six alkaloid contents in each batch.

## Supplementary Materials

The Supplementary Materials are available online.
