# Supplementary material for: Quality Assessment of Kumu Injection, a Traditional Chinese Medicine Preparation, Using HPLC Combined with Chemometric Methods and Qualitative and Quantitative Analysis of Multiple Alkaloids by Single Marker
Source: Molecules. 2018 Apr 9;23(4):856. doi: 10.3390/molecules23040856 (PMC6017852; doi:10.3390/molecules23040856)
Supplement: Supplementary file 1 [file molecules-23-00856-s001.pdf]

Supplementary

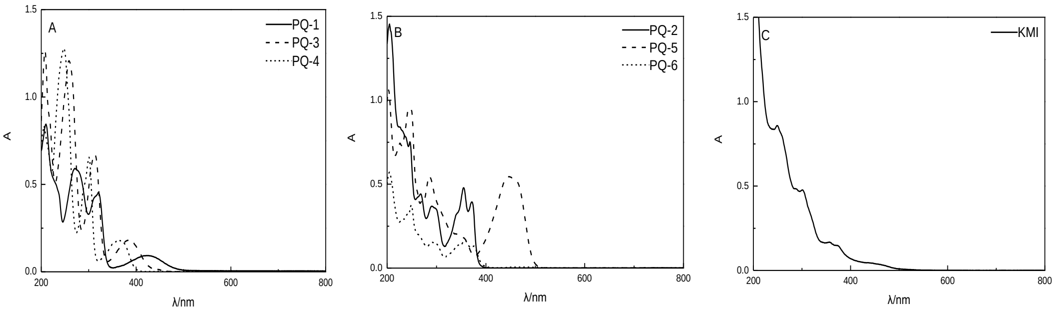

Figure S1. UV spectrograms of the *β*-carboline alkaloids PQ-1, 3, 4 (A), the canthinone alkaloids PQ-2, 5, 6 (B), and KMI (C)

Table S1. Results of the system suitability test for QAMS method [1].

Analytes		Inject No.						Mean	RSD/%
		a	b	c	d	e	f		
PQ-1	T	1.16	1.16	1.21	1.26	1.31	1.02	1.19	8.5
	n	29554	28735	30330	31847	31666	32136	30711	4.5
	R	29.62	28.99	29.21	29.87	30.31	30.26	29.71	1.8
	SNR	1815	2452	2003	2008	2439	1958	2112	12.6
PQ-2	T	1.03	1.01	1.01	1.01	1.04	1.05	1.03	1.7
	n	739892	758489	741164	742110	742050	741740	744241	0.9
	R	19.69	20.07	19.84	19.78	19.25	19.34	19.66	1.6
	SNR	1162	1570	1291	1313	1610	1286	1372	12.9
PQ-3	T	1.04	1.15	1.05	1.06	1.07	1.09	1.08	3.7
	n	86821	67447	69647	80644	88953	92607	81020	12.9
	R	5.81	6.06	6.29	6.41	6.41	6.43	6.24	4
	SNR	6151	7324	6095	6675	8756	7191	7032	14
PQ-4	T	0.79	0.79	0.80	0.81	0.83	0.84	0.81	2.6
	n	52788	51185	51737	51260	52204	51763	51823	1.2
	R	1.54	1.50	1.51	1.60	1.62	1.71	1.58	5
	SNR	3276	4436	3686	3655	4448	3552	3842	12.7
PQ-5	T	0.93	0.92	0.94	1.00	1.03	1.05	0.98	5.7
	n	51507	49613	48823	49747	49095	49383	49695	1.9
	R	8.99	9.20	9.14	9.21	8.95	8.6	9.02	2.6
	SNR	978	1331	1082	1066	1287	1028	1128	12.8
PQ-6	T	0.95	0.96	0.96	0.95	0.88	1.09	0.97	7.1
	n	636333	635926	620070	637503	654769	637821	637070	1.7
	R	20.19	19.75	19.88	20.13	20.19	20.08	20.04	0.9
	SNR	4807	6458	5283	5391	6653	5323	5652	12.9

[1] *T*-tailing factor; *n*-theoretical plate number; *R*-resolution; *SNR*-signal to noise ratio.

Table S2. Samples information [2].

Code No. a)	Batch No. b)	Code No. a)	Batch No. b)	Code No. a)	Batch No. b)
S1	2014111103	S8	2015090403	S15	2016040703
S2	2014122503	S9	2015121103	S16	2016040803
S3	2014122803	S10	2016021903	S17	2016040903
S4	2014123003	S11	2016022003	S18	2016041003
S5	2015010503	S12	2016022103	S19	2016072303
S6	2015030203	S13	2016040503	S20	2016072403
S7	2015071203	S14	2016040603	S21	20160520

[2] a) S1 through S20 were Kumu injections (KMI) registered as Hailixin® and manufactured by Jiangxi Qingfeng Pharmaceutical Co., Ltd (Ganzhou, China); S21 was negative preparations (NP) supplied by Jiangxi Qingfeng Pharmaceutical Co., Ltd (Ganzhou, China); b) The first six digits in batch number of S1 through S20 represented the production date.

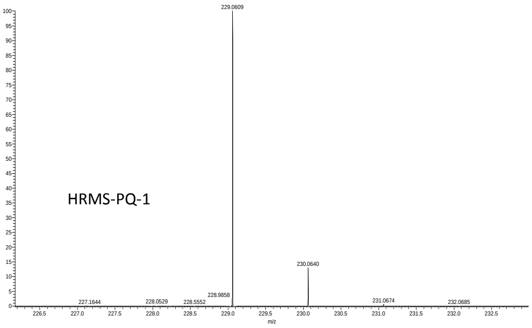

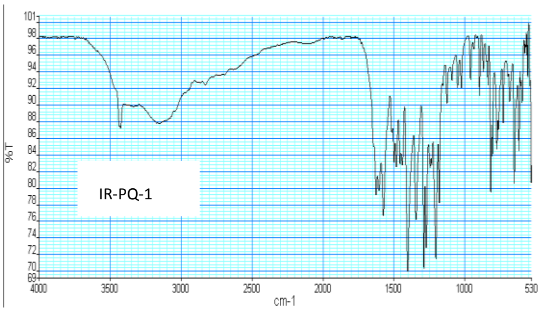

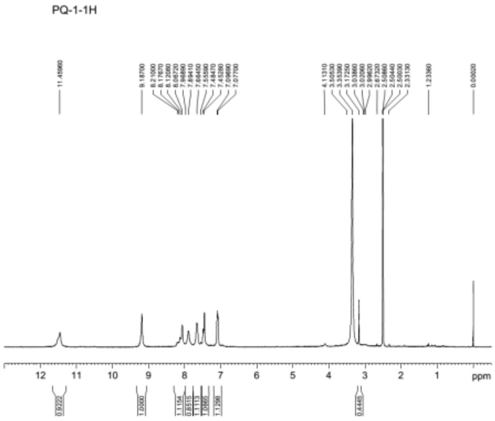

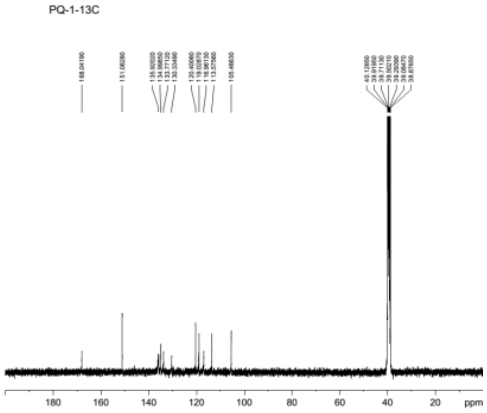

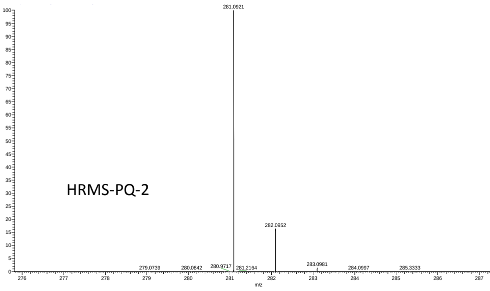

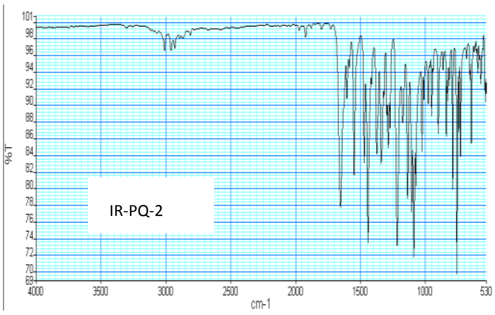

PQ-2-1H

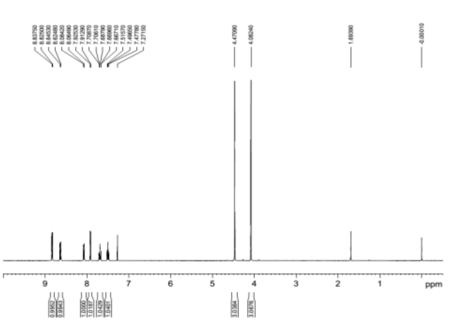

PQ-2-13C

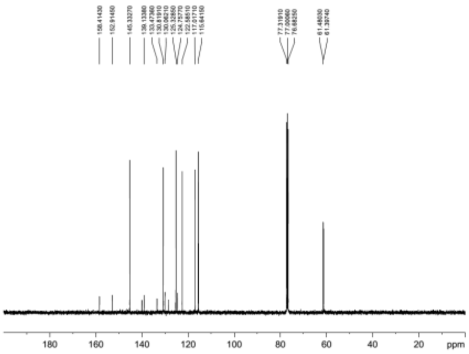

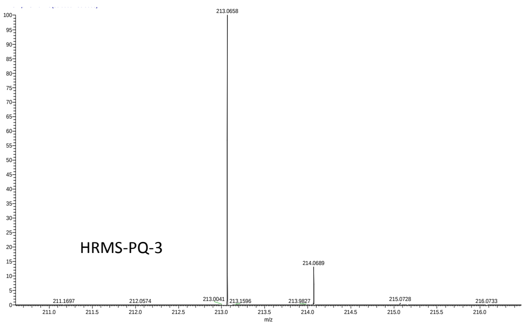

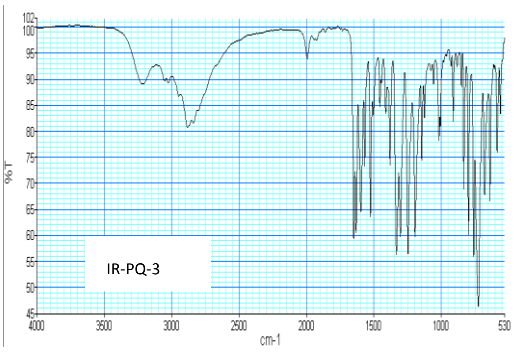

PQ-3-1H

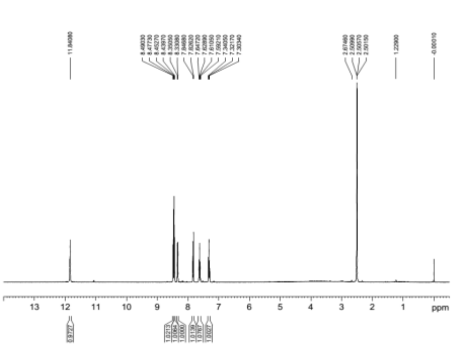

PQ-3-13C

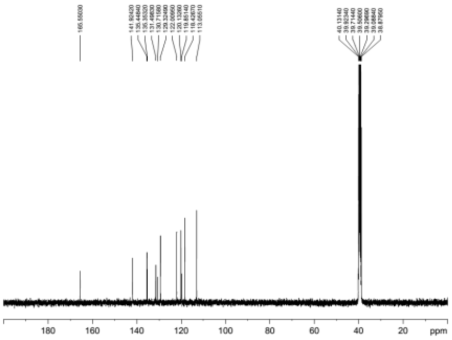

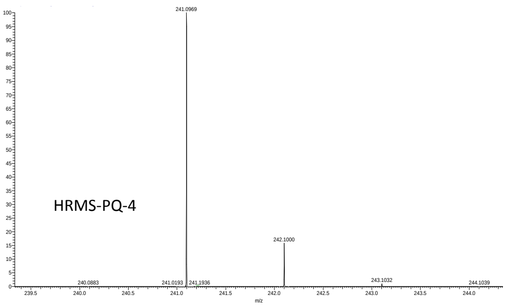

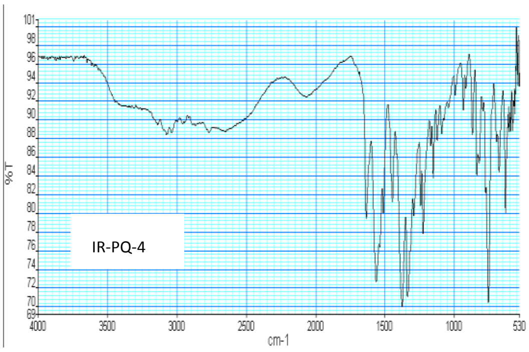

PQ-4-1H

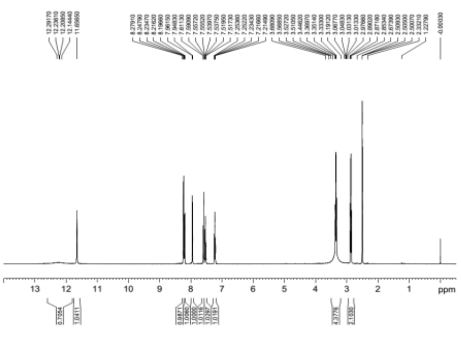

PQ-4-13C

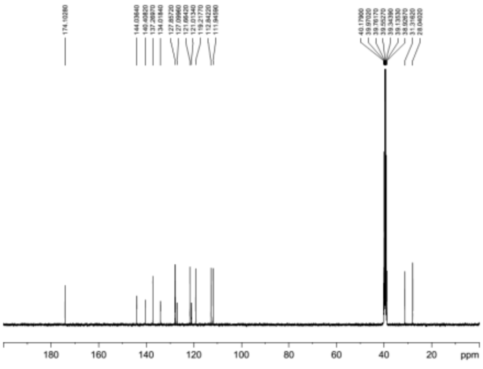

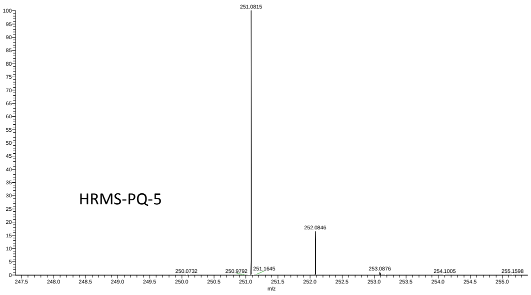

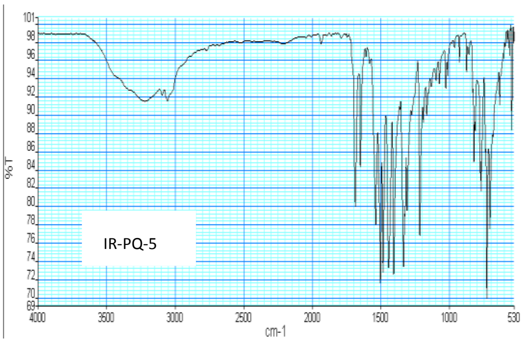

PQ-5-1H

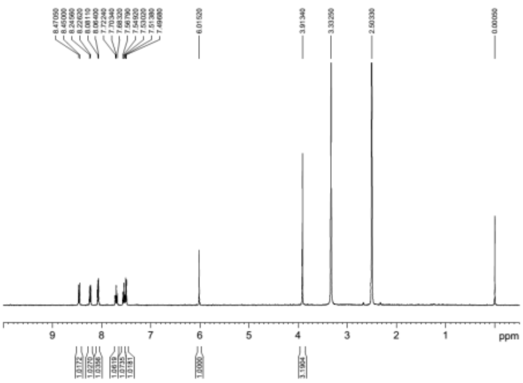

PQ-5-13C

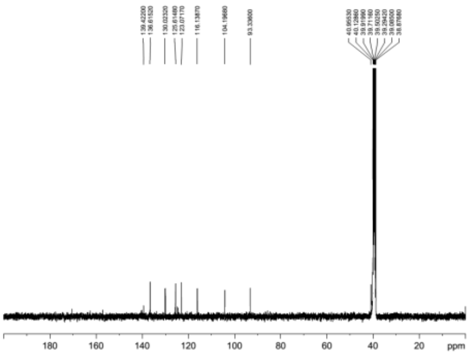

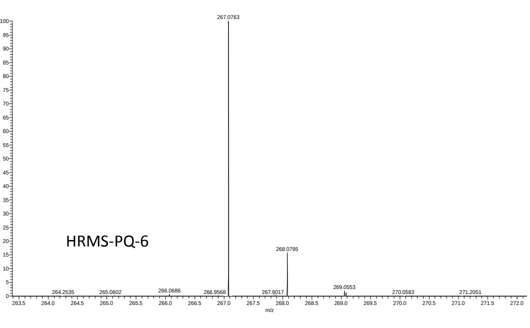

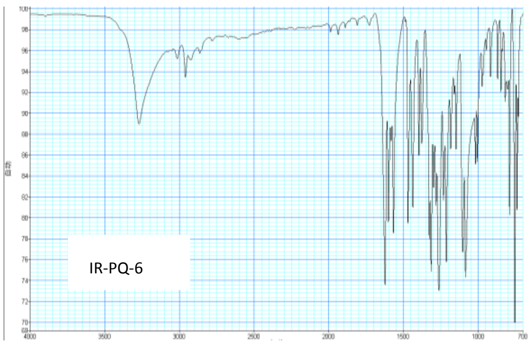

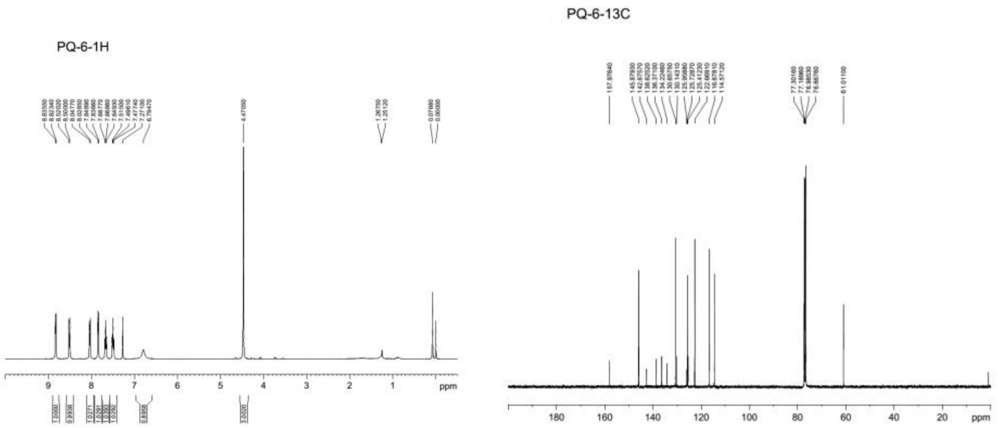

Figure S2. Comprehensive spectroscopic analysis

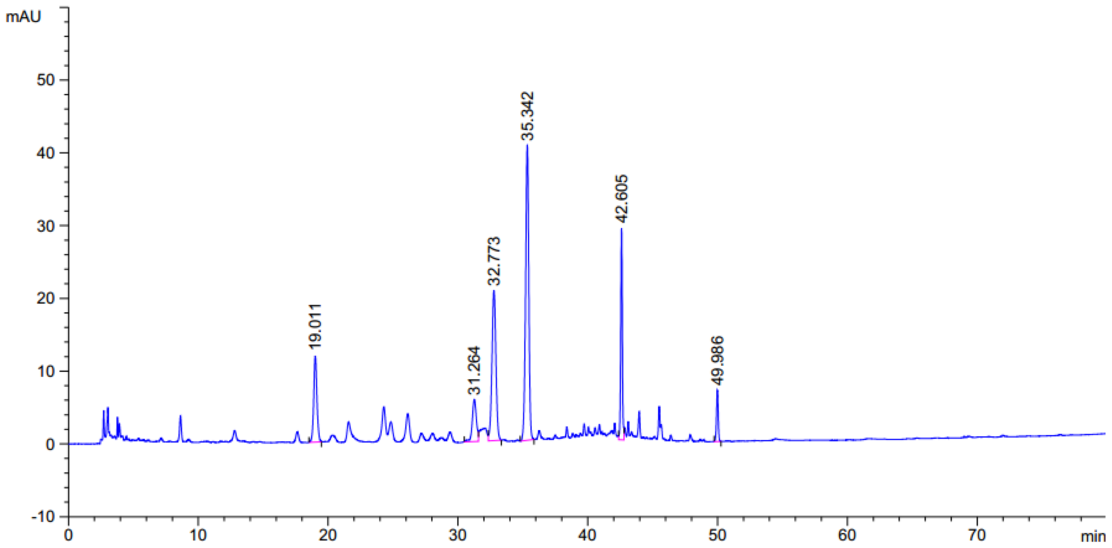

Figure S3. An 80 min chromatogram of KMI
